# Space Occupying Lesions in the Fetal Chest Evaluated by MRI

**DOI:** 10.5812/iranjradiol.3934

**Published:** 2012-09-17

**Authors:** Umit Aksoy Ozcan, Ersan Altun, Latif Abbasoglu

**Affiliations:** 1 Department of Radiology, School of Medicine, Acibadem University, Istanbul, Turkey; 2 Department of Pediatric Surgery, Acibadem, Istanbul, Turkey

**Keywords:** Magnetic Resonance Imaging, Congenital diaphragmatic Hernia, Cystic Adenomatoid Malformation of Lung, Congenital, Bronchopulmonary Sequestration

## Abstract

**Background:**

The most common space occupying lesions of the fetal thorax are congenital diaphragmatic hernia (CDH), congenital cystic adenomatoid malformation (CCAM), and bronchopulmonary sequestration (BPS). Although applications of prenatal MRI have been vastly improved in the recent years, its use in the assessment of space occupying lesions of the fetal chest differs among centers.

**Objectives:**

To evaluate MRI findings in the diagnosis and follow-up of space-occupying lesions in the fetal chest with the review of relevant literature.

**Patients and Methods:**

The fetuses with space-occupying lesions of the chest were retrieved from our 1.5T fetal MRI database of 347 patients. MRI features including the shape, signal characteristics, feeding artery, margin, mass effect, affected organ parts and anatomic location were reviewed. The results were correlated with the pathology results, follow-up and surgical findings.

**Results:**

Nineteen MR images of 17 fetuses (mean gestational age, 23.8 weeks) with spaceoccupying lesions (5 CCAMs including one involuted case), 2 BPSs, 2 hybrid lesions, 8 CDH) were evaluated. One case of CCAM completely involuted in utero, four newborns were operated, and the resulting 12 fetuses were terminated. The surgical and pathological findings were in accordance with MRI findings.

**Conclusion:**

MRI can reliably differentiate CDH from CCAM and BPS in utero. Follow-up is of utmost importance as lesions may involute or progress in utero. Prenatal MRI findings help postnatal decision-making, surgical planning and parental counseling.

## 1. Background

The ability to identify and diagnose the congenital fetal lesions has increased in the recent years with the emergence of ultrafast MRI sequences. The most common masses seen in the fetal chest are congenital diaphragmatic hernia (CDH), congenital cystic adenomatoid malformation (CCAM), and bronchopulmonary sequestration (BPS) (1). During the course of pregnancy, space-occupying lesions may enlarge rapidly or grow commensurately with the fetus. Rarely, CCAM and BPS may partially or completely involute (1). MRI has become a complementary tool to ultrasonography (US) in the evaluation of space occupying lesions in fetal chest because of its exquisite delineation of the airway and lung parenchyma (2, 3). Preoperative reliable differential diagnosis is especially important in congenital chest masses where immediate medical and/or surgical intervention is required in the early postnatal period. However, the use of MRI in the assessment of space occupying lesions in the fetal chest differs among centers.

## 2. Objectives

The purpose of this study is to evaluate the role of MRI in the diagnosis and follow-up of space occupying lesions of the fetal chest with the review of relevant literature.

## 3. Patients and Methods

Between June 2008 and January 2011, a total of 347 patients suspected of having abnormal fetuses on obstetric US were referred for further assessment with MRI. Among these, the fetuses with space-occupying lesions of the chest were retrieved from the database. In our institution, written informed consent is obtained before fetal MRI and no sedation is given to the mothers. The institutional ethics committee had approved this retrospective study.

MR imaging was performed with a 1.5-T machine (Magnetom Symphony; Siemens, Erlangen, Germany) by using a receive-only CP body-array coil with the patient in the supine position. All patients were examined with our routine fetal MRI protocol including half-fourier acquisition single shot turbo spin-echo (HASTE) and/or true fast imaging with steady state precession (True FISP) and 2D fast low angle shot (FLASH) T1 weighted sequences. The images were obtained in fetal orthogonal planes with the following parameters; one signal acquired, slice thickness:3-4mm, no intersection gap, rectangular field of view matrix: 240 × 256, and field of view: 26-35 cm (optimized for gestational week (GW) of each fetus), acquisition: 1. The other imaging parameters were as follows; HASTE: TE: 78 msec, TR:1000 msec, flip angle: 150, ETL: 179, bandwidth: 230 Hz/pixel; true FISP: TE: 2.3 msec, TR:4.6 msec, flip angle: 80, bandwidth: 501 Hz/pixel; 2D FLASH T1: TE: 166 msec, TR: 4.76 msec, flip angle: 70, bandwidth: 110 Hz/pixel. The total acquisition time was no more than 20 seconds for each sequence and 20 min for each fetus. All images were evaluated by one observer with 10-years experience in fetal MR interpretation. The various features were analyzed including the shape of the lesion, signal characteristics, the feeding artery (signal-void structure), margins, mass effect (mediastinal compression), affected organ parts, anatomic location, involutional changes in the follow-up of patients and additional abnormalities. CCAM lesions were categorized according to Stocker et al. (3-10cm large cysts: type 1, numerous small cysts: type 2, microcystic cysts (0.2 cm): type 3) (4). Fetuses that were not terminated were followed up with US, and MRI, and after delivery, neonatologists examined the infants. Pediatric surgeons operated live born fetuses with CDH and selected cases of CCAM/BPS. MRI findings were correlated with the post-operative findings and pathology results as the gold standard in all cases.

## 4. Results

**Figure 1:**
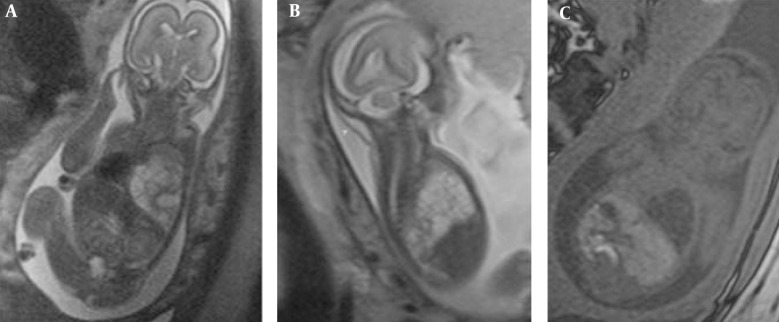
A 20-week-old fetus with CCAM type 1 of the left hemithorax. Note the intact diaphragm and mediastinal shift to the right on coronal HASTE images (A) associated with mild hydrops observed on the sagittal image (arrow) (B). T1W coronal images show hypointense CCAM and hyperintense liver (C).

In the retrospective analysis, 19 consecutive MRI exams of 17 fetuses [mean gestational week (GW), 23.8 weeks; range, 20-31weeks] with space occupying lesions of the chest were retrieved from the database constituting 4.9% (17/349) of the fetuses referred for MRI in our institution (Table 1). The mothers were referred to the MRI section for the following indications: limited ultrasound evaluation (maternal obesity, twin pregnancy) (3), multiplanar assessment of involved organ systems in a complex anomaly (5), assessment of extrapulmonary diseases (2), referral from other medical facilities with equivocal findings on US (6) and finally for follow-up (2). CCAM was diagnosed on the basis of MR findings in five fetuses (including one fetus with the involuting lesions), BPS in two fetuses and hybrid lesions in two fetuses. In type 1 CCAM cases, multiple cysts were observed and the largest observed cyst was 23 mm. The lesions had high signal intensity on HASTE images almost equal to amniotic fluid and markedly higher than the adjacent lung tissue. Contours were lobulated, but well demarcated from the adjacent lung tissue. No feeding arteries were evident in CCAM cases. In all cases, the diaphragm was intact and well delineated. In case no. 3, the mediastinum and heart were displaced towards the right hemithorax and the lesion occupied almost two thirds of the fetal thorax causing mediastinal shift and hydrops (Figure 1 A-C).

**Table 1:** Detailed Information for Each Fetus Regarding the Gestational Weeks, MRI Diagnosis and Post-Surgical and/or Histopathological Diagnosis

**GA**	**Findings**	**Prognosis**
24	RLL a CCAM a type 1	Termination/pathology: type 1 CCAM
23	RLL CCAM type 1	Operated in postnatal period, type 1
20	RLL CCAM type 1 and Mediastinal shift, hydrops	Termination/pathology: type 1 CCAM
22	LLL a CCAM type 2	Termination/pathology: type 2 CCAM
24	RLL- BPS a and high arched palate	Termination/pathology: BPS
24	RLL- CCAM type 2 and BPS (hybrid lesion), feeding artery (+), left lung SI increased	Termination/pathology: Hybrid lesion
24	RLL hybrid (CCAM type 2+BPS), mediastinal shift, polyhydram niosis	Termination/pathology: Hybrid lesion
22	RLL-posterior BPS, mild mediastinal shift	Termination/pathology: BPS
25-31	RLL and RUL CCAM type 3	Complete prenatal involution at 31^st^ GW a-CXR and 2 years follow-up normal
20	L CDH a (stomach, intestines), mediastinal shift	Termination/pathology: CDH
20	L CDH (stomach, intestines) and Trisomy 18	Termination/pathology: CDH, Trisomy 18
30	L CDH (stomach, intestines, spleen)	Postnatal hernia repair
22-33	Twin, L CDH (stomach, intestines, colon, liver) polyhydramniosis of the fetus with CDH	MRI follow-up and postnatal hernia repair
35	L CDH (stomach, intestines, left lobe of the liver) and Arnold Chiari type 2 malformation	Termination/pathology: CDH and ACH II
20	L CDH (stomach, intestines, left lobe of liver, spleen) and poly-hydramniosis, left ventricle and left lung hypoplasia	Termination/pathology: CDH
35	L CDH (stomach, intestines, spleen) and L mild hydronephrosis	Postnatal hernia repair
23	L CDH a(stomach, intestines, left kidney)	Termination/pathology: CDH

^a^Abbreviations: ACH; Arnold Chiari, BPS, bronchopulmonary sequestration; CCAM, congenital cystic adenomatoid malformation ; CDH, congenital diaphragmatic hernia, GW; Gestational week, LLL; left lower lobe, RLL; right lower lobe, SI; Signal intensity

**Figure 2:**
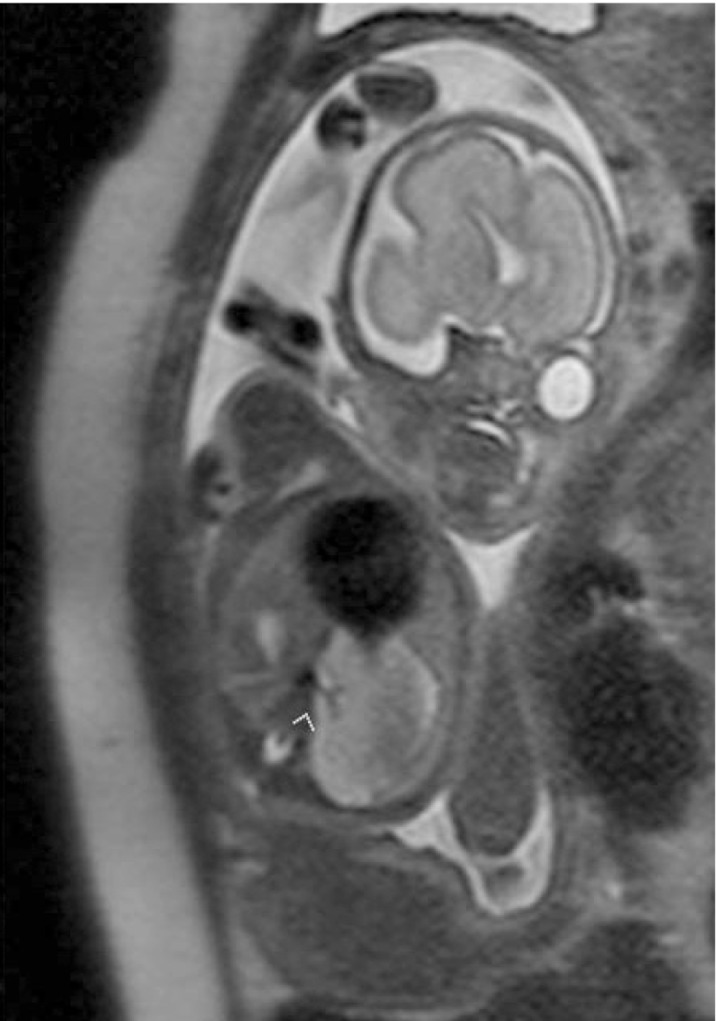
A 24-week-old fetus with BPS on the right side Homogeneous hyperintense lesion with regular contours with direct branching vessel from the thoracic aorta can be observed on axial HASTE image (arrow).

**Figure 3:**
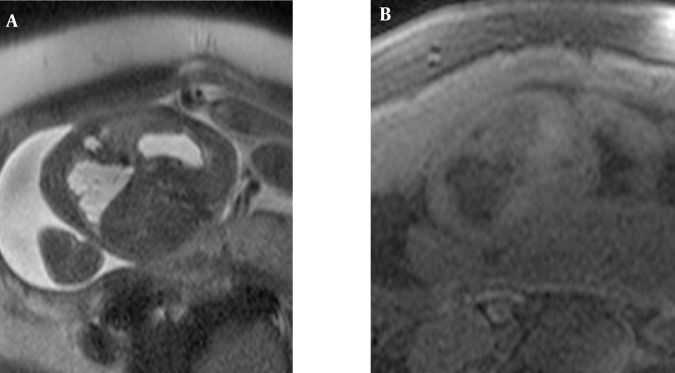
A 24-week-old fetus with hybrid lesion of the right lung Peripheral small cysts with higher signal intensity than the central part and lobular contours are observed on axial HASTE images (A), and corresponding T1W images exhibit hypointense lung lesion with more hypointense tiny peripheral cysts (B).

MRI demonstrated feeding arteries arising from the aorta in all four fetuses with BPS (pure or hybrid), although the venous drainage into the pulmonary veins could not be visualized. In the two cases with pure BPS, the MRI appearance was of a well-defined, triangular, homogeneous, hyperintense mass (compared to normal lung) with a signal-void feeding artery (Figure 2). Two hybrid cases with BPS and type 2 CCAM had lobulated margins and inhomogeneous hyperintensity with a signal-void feeding artery. Peripherally located cysts up to 12mm were observed (Figure 3 A-B). On T1W images, the liver signal was higher than the lung facilitating the contour delineation. On T1W images, CCAM lesions were hypointense almost equal to the amniotic fluid, on the other hand BPSs were also hypointense relative to the lung, but pure BPS had higher signal intensity than CCAM. In hybrid cases, the small cysts were observed more hypointense than the central BPS component.

**Figure 4:**
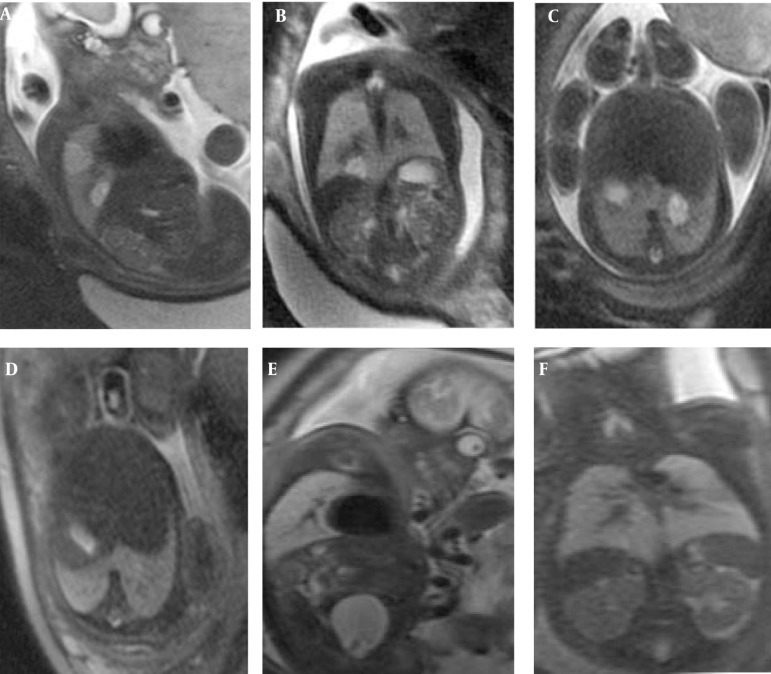
Well circumscribed homogeneous hyperintense right upper and lower lobe lesions are observed at 25th GW on sagittal and coronal HASTE images (A,B) and follow-up at 31st GW both lesions involuted completely (C,D). The lower lobe lesion at 25th GW (E) and involuted at 31st week (F) on axial images.

**Figure 5:**
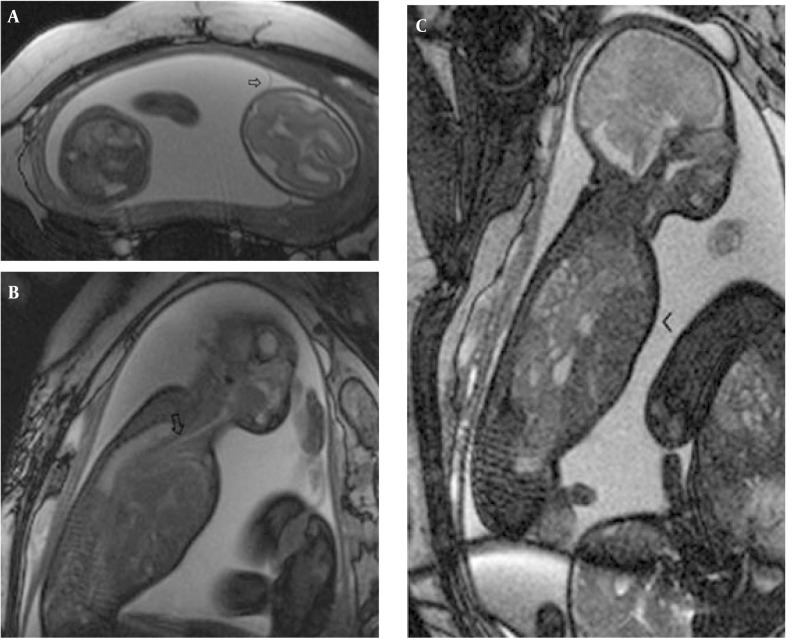
Twin gestation at the 22nd GW Polyhydramniosis of the amniotic sac of the fetus with CDH is observed on the axial image (arrow) (A); on follow-up images at the 33rd GW left CDH compressing the esophagus (open arrow) (B), and herniated liver (arrowhead) can be observed on sagittal images (C).

**Figure 6:**
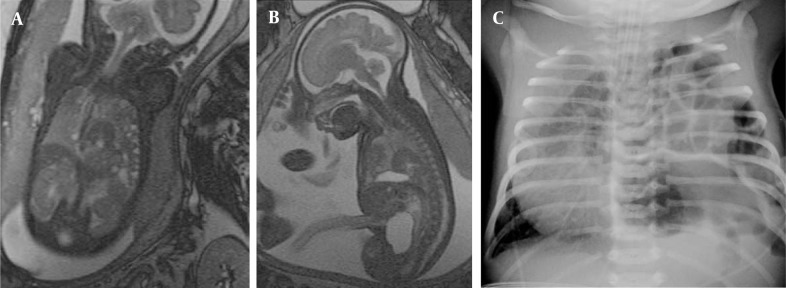
Congenital diaphragmatic hernia in the 35th GW fetus including intestines and spleen on, coronal (A) and sagittal images (B), and postnatal chest x-ray (C).

In one fetus (case no. 9) well-delineated right upper and lower lobe hyperintense lesions without observed feeding arteries were detected at the 25th GW and follow-up was recommended. At the 31st GW, both lesions involuted completely and the delivery was uneventful (Figure 4 A-F). The postnatal chest x-ray was normal and he is 2-year-old now, clinical follow-up is normal without any respiratory problems. One fetus with CCAM (case no. 2) was delivered in another center and referred to our neonatal intensive care unit with severe respiratory problems when he was 2-days old. After the infant was transferred to our facility, the fetal MRI diagnosis of CCAM was achieved. Immediate operation was planned based on fetal MRI images and the infant was operated without further imaging and was stable in the postoperative period. The remaining seven pregnancies were terminated based on parental decision. In eight fetuses, CDH was diagnosed on the basis of MR findings. In all CDH cases, the stomach and intestines were partially or completely located in the left hemithorax. Other herniated organs were the liver in three cases (case no. 13, 14, 15), the spleen in two cases (case no. 15 and 16) and the left kidney in case no. 17. On T1W images, the herniated liver was easily observed since it has higher signal intensity than the other herniated organs. Five cases were terminated and three were operated in the postnatal period. One of the mothers who was imaged at the 22nd GW had twin fetuses; one of them had CDH and the other was normal. The amniotic sac of the fetus with CDH was bulging indicating polyhydramnios. Follow-up MRI was performed on the 33rd GW and esophageal compression by the herniated organs and mediastinal shift was clearly visible (Figure 5 A-C). In three cases, operations were planned based on prenatal MRI images and only chest x-rays were obtained before surgery (Figure 6 A-C). In four operated newborns (three CDHs and one CCAM), the operation findings were in accordance with prenatal diagnosis. The resulting 12 cases were terminated upon parental request and the pathology results were in accordance with the preoperative diagnosis.

## 5. Discussion

In cases of intrathoracic space occupying lesions, survival after birth is greatly dependent on adequate assessment of the lesion and development of pulmonary hypoplasia in the antenatal period. Our study demonstrated that the differential diagnosis of the most common three fetal chest lesions; namely, CCAM, BPS and CDH may reliably be made by fetal MRI and follow-up MRI has utmost importance since the lesions may completely involute or progress to exhibit additional complications such as hydrops and mediastinal shift. Accurate diagnosis supports parental decisions about the course of pregnancy by differentiating the lesions with a possibility of involution such as CCAM and BPS from CDH that requires postnatal surgery. Besides, fetal MRI can accurately guide the postnatal surgery by depicting all herniated organs including the thoracic kidney in CDH and identifying the extent of disease in CCAM, obviating the necessity for extensive postnatal imaging.Accurate prenatal diagnosis of chest lesions is important because the natural history of these lesions and their treatment vary substantially (1). On US, the classic differential diagnosis for an echogenic lung mass is CCAM, sequestration, or CDH. Accompanying anechoic structure may be the stomach, intestines or cyst, and sometimes the systemic blood supply is visualized (5). However, sonographic differential diagnosis is not always possible and fetal MR imaging can be helpful when the diagnosis is unclear by easily differentiating CDH from CCAM and BPS (1, 3).

CCAM is a rare lesion with a prevalence of 9:100,000 total births (6, 7). It is characterized by a multicystic mass of pulmonary tissue with abnormal proliferation of bronchiolar structures that connect to the normal bronchial tree and also called as congenital pulmonary airway malformation (1, 5, 8). The vascular supply of CCAM is from the pulmonary artery with drainage into the pulmonary veins. Hence, the vascular supply of classical CCAM is not observed in any of our cases with MRI. BPS is generally thought to result from an abnormal accessory tracheobronchial bud arising from the foregut accounting for up to 6% of congenital lung malformations (9, 10, 11). Sequestration may be extralobar (more diagnosed in the prenatal and neonatal period) and intralobar (more diagnosed in the childhood period) (1). It has its own vascular supply from the systemic circulation and if a vessel arises from the aorta, the lesion is presumed to be a sequestration. The vasculature supplying BPSs were visualized on MRI in all our cases including the hybrid cases. However, there is a wide spectrum of these anomalies with much overlap. Stocker et al. (4) categorized CCAM into three histologic types; type 1 consists of large cysts (3-10 cm), type 2 numerous cysts smaller than 2cm and type 3 is microcystic lesions (0.2cm) that appears as a solid mass (12). Although MR characteristics are known for the initial three types, type 0 and type 4 were later added to the classification (13). On MR imaging, CCAM and BPS exhibited higher signal intensity than normal adjacent lung tissue on T2-weighted imaging and lower signal intensity than normal lung on T1-weighted imaging. Type 1 and type 2 CCAM are very high signal on T2-weighted images almost equal to amniotic fluid; however, the signal intensity of BPS was not that high, yet still higher than the lung tissue. Type 3 CCAM is relatively homogeneous and has moderately high signal intensity that is higher than that of normal lung, but not as high as that of the amniotic fluid. On MRI, pure fetal BPS appears as a well-defined, triangular, homogeneous and hyperintense mass (5). In our cases, isolated BPSs had well-defined contours, but hybrid lesions contained peripheral small cysts causing irregular contour appearance. The hybrid cases had feeding vessels from the aorta. Sequestrations are frequently located in the lower lobes so were our cases. One of the CCAMs in this series developed hydrops because of the mediastinal shift. The prognosis of CCAM lesions is variable, depending on the size rather than the histological type (1). If large, they can cause mediastinal shift, pulmonary hypoplasia, vascular compromise and hydrops. Hydrops is a harbinger of impending fetal death and this fetus was terminated. When adjacent normal lung is compressed by a pulmonary mass, such as a CCAM or sequestration, it can be visualized on MR as of slightly lower signal intensity than adjacent normal lung. In addition, polyhydramniosis may develop due to impaired swallowing, caused by esophagus compression by the mass. Although rare, CCAM and BPS may involute partially or completely (1, 14, 15). Even rarer, complete in utero regression may be observed. In one of our cases with two small pulmonary hyperintense lesions that may indicate CCAM type 3, the lesions completely disappeared in utero in the follow-up period. The lesions may disappear on sonography, but still be visible on MR imaging. Liu et al. state that other than large CCAMs, all CCAMs showed some degree of regression in their series (2). As CCAM and BPS lesions regress, their signal intensity decreases. Partially regressed BPS tends to have a lobulated margin with a decrease in signal intensity and signal inhomogeneity. Levine (5) et al. found that larger lesions did not regress as much as smaller ones. A pleural effusion may be visualized transiently as the lesion decreases in size, but this was not evident in our case.

Congenital diaphragmatic hernias occur in approximately 1 in 2,200–12,500 live births and typically occur in the posterolateral left hemidiaphragm (16). The reported cases in this series were also located on the left side. Right-sided, bilateral, paraesophageal and pericardial hernias may also occur (5). The classic US signs are cystic thoracic lesion with a mediastinal shift and occasionally an absence of a normally positioned fluid- filled stomach (17). However, these sonographic findings do not always warrant a sufficient differential diagnosis and MR may be used for clarification of the findings. The contours of the diaphragm may easily be traced in the sagittal and coronal MR images. Besides, herniated organs like stomach, intestines, spleen and the liver can be identified by their characteristic MR appearances (18, 19). In this series, herniated liver and spleen were seen in 50% of the CDH cases. The liver is observed on MR imaging as a slightly low signal intensity structure on T2-weighted imaging that is of higher signal intensity on T1-weighted imaging. The high morbidity associated with CDH is due to pulmonary hypoplasia resulting from the compression of the developing lungs by the herniated viscera (5, 20). For diaphragmatic hernias in general, the position of the liver (above or below the diaphragm) is one of the best positive predictive values for survival, especially liver in the chest is associated with a worse prognosis than when the liver is completely intra-abdominal (5, 21). Colon, with a high signal intensity on T1-weighted imaging and low signal intensity on T2-weighted imaging, the small bowel with fluid-filled loops, the stomach and the spleen, all can be well-visualized in hernias (5). In this series, one case (case no. 17) was reported to have an intra-thoracic kidney. The incidence of intra-thoracic kidney as a result of congenital diaphragmatic hernia is extremely rare with a prevalence of less than 0.01% (20, 22, 23). Incorrect prenatal diagnosis may have poor clinical results as many infants with space-occupying lesions suffer from respiratory problems. Fetal MRI may obviate postnatal CT or MRI necessity, which requires sedation to some extent especially when performed in the late gestational period. Our CDH cases no. 12, 13, and 16 were operated with prenatal planning. Termination of fetuses with US diagnosis of space-occupying lesions of the chest limits the number of fetuses referred for prenatal MRI. The assessment of space-occupying lesions of the chest with larger series would provide additional information of involuting lesions as well as providing guidance for postnatal surgery.

In conclusion, fetal MRI is helpful for reliable differential diagnosis of most common space occupying lesions of the chest. Follow-up MRI is important since the lesions may completely involute in utero or may be complicated by hydrops and mediastinal shift. Accurate differential diagnosis enabled by the multiplanar capability of MRI may help parental counseling; moreover, this may guide the immediate postnatal surgery obviating the necessity for extensive postnatal imaging.
